# Energy Intake and Severity of Dementia Are Both Associated with Health-Related Quality of Life among Older Long-Term Care Residents

**DOI:** 10.3390/nu11102261

**Published:** 2019-09-20

**Authors:** Salminen KS, Suominen MH, Kautiainen H, Roitto HM, Pitkala KH

**Affiliations:** 1 University of Helsinki, Department of General Practice and Primary Health Care, POB 20, 00014 Helsinki, Finlandhannu.kautiainen@medcare.fi (K.H.);; 2 Vantaa Social Welfare and Health Care, POB 1600, 01030 Vantaa, Finland; 3 Department of Social Services and Health Care, Geriatric Clinic, Helsinki Hospital, POB 6600, 00099 Helsinki, Finland; 4 Helsinki University Hospital, Unit of Primary Health Care, 00014 Helsinki, Finland

**Keywords:** energy intake, health-related quality of life, stage of dementia, long-term care

## Abstract

Our aim was to investigate how energy intake modifies the association of the stage of dementia with health related quality of life (HRQoL) among institutionalized older people. A cross-sectional sample of 538 older long-term care residents with dementia in Helsinki, Finland were assessed with HRQoL (15D), energy intake (from one to two days), and the stage of dementia by the clinical dementia rating (CDR) scale. The energy intakes were standardized by *z*-scores to include both men and women in the same analyses. Severity of dementia was associated with HRQoL (15D index in CDR 0.5–1: 0.65 (0.11), CDR 2: 0.60 (0.10), CDR 3: 0.52 (0.10)). When the three groups of dementia severity were divided according to their energy intake quartiles, there was an association between the HRQoL and the stage of dementia (*p* < 0.001) and energy intake (*p* = 0.013); however, no interaction was observed (*p* = 0.30). While partial correlation analysis showed that energy intake correlated with HRQoL among residents with very mild/mild or moderate dementia, this was not observed among those with severe dementia. In moderate dementia, the dimensions of mobility and usual activities correlated significantly with higher energy intake. Both energy intake and severity of dementia are associated with HRQoL.

## 1. Introduction

When measured by the Mini-Nutritional Assessment (MNA), the prevalence of malnutrition in nursing homes in Europe ranges from 14–21% and from 21–40% in long-term care [1]. However, in an earlier study on a heterogeneous sample of older people whose nutritional status was normal according to the MNA, only approximately 40% of participants reached the amount of energy and 25% reached the amount of protein recommended by the Nordic Nutrition Recommendations [2,3]. Engelheart et al. observed that there was no correlation between nutritional status and energy or protein intake among older people living at home or in a nursing home [4]. Thus, assessing energy and protein intake might be a more sensitive assessment than a mere MNA screening tool to detect those at risk of developing malnutrition [2,4,5].

The energy and protein intake in institutionalized older people varies between studies depending on culture, dementia stage, age, and gender distribution. A Dutch study showed that only 18% of residents from a nursing home rehabilitation ward received adequate energy and protein intake [6]. In this Dutch study (*n* = 79), protein intake was considered sufficient if the resident reached ≥ 1.2 g/bodyweight, and energy intake was considered sufficient if the resident reached ≥ 85% of their energy need, as calculated by Harris and Benedict + 30%. In a Swedish study (*n* = 128), 16% of the residents living in a nursing home were malnourished according to the MNA, and received a mean energy intake of 1724 kcal/day and mean protein intake of 63 g/day [4]. A study on Finnish residents (*n* = 374) from assisted living facilities revealed a mean energy intake of 1762 kcal/day and a protein intake of 64 g/day; 17% of residents were malnourished according to MNA [7]. In a recently published Spanish study among nursing home residents (*n* = 249), the mean energy intake was 1624 kcal/day, protein was 60 g/day, and 17% of the residents were malnourished according to MNA short-form [5]. Previous studies on long-term care have shown that especially residents with severe dementia and disabilities are at risk for malnutrition [2,8]. Low energy intake has been associated with sarcopenia [5,9] and frailty [10].

Health-related quality of life (HRQoL) should be considered a goal of good aging [11]. HRQoL is a multidimensional concept that includes an individual’s perceived physical and mental health. Quality of life (QoL) is a broader concept; in addition to health and functioning, it includes psychological, social, and environmental dimensions [12].

In some studies, QoL decreased over follow-up time, while in others, QoL was unchanged or even increased among institutionalized persons with dementia [13,14,15,16,17]. These studies differ from each other in follow-up time, methods used to evaluate QoL, and settings. This may explain the differences between studies. Thus, there are contradictory findings concerning how HRQoL is associated with the stage of dementia among institutionalized older residents.

Malnutrition is associated with poor HRQoL among non-institutionalized older people [11,18,19,20,21,22]. Physical performance and functioning declines with malnutrition, and this decline may also impair HRQoL [21]. Wei et al. observed that nutritional deterioration is associated with poor QoL during follow-up among community-living older people [23]. In a previous study, increased energy intake is associated with better HRQoL among non-institutionalized older people and home-care patients [11,24]. Studies investigating the association between energy intake and HRQoL in different stages of dementia are scarce.

Nutrition and food intake are important aspects of QoL among residents in nursing home settings. Nutritional status is an important aspect of HRQoL and psychological well-being [22,25,26]. However, less is known about how nutrient intake is associated with HRQoL. To our knowledge, there are no studies that have explored how energy and food intake modify HRQoL in various stages of dementia. The aim of this study was to determine how dementia severity is associated with HRQoL and how nutrient intake modifies this association.

## 2. Material and Methods

### 2.1. Study Sample

This was a cross-sectional study designed to assess various aspects of nutrition among older residents in institutional settings in Helsinki, Finland. The study aimed to obtain a comprehensive picture of nutritional status, nutrient intake, nutritional care, and related factors in institutional settings. The participants were recruited from three nursing homes and 14 assisted living facilities in Helsinki. Although the level of care is basically the same in nursing homes and in assisted living facilities, the residents are more mobile in the latter. In assisted living facilities, residents have their own room and furnishings, while in nursing homes, residents have shared rooms. For residents with dementia, there are group homes in assisted living facilities. Continuous assistance is available and a registered nurse is in charge of the ward.

The inclusion criteria for the present study were: aged ≥ 65 years; native Finnish or Swedish speaker (all participants were Caucasian); living permanently in institutional care; sufficient information on demographic factors; and assessed with a clinical dementia rating (CDR), a one to two day food record, and HRQoL according to the 15D instrument. Altogether 538 residents met these criteria and were included.

The local ethics committee of the Helsinki University Hospital approved the study (No. HUS/2042/2016). Informed consent was acquired from all participants or from their closest proxies in case of moderate-severe dementia (Mini-Mental State Examination (MMSE) < 20 points).

### 2.2. Measurements

Trained nurses collected the data. The resident’s demographic information, diagnoses, and current use of medications were retrieved from medical records. The Charlson Comorbidity Index (CCI) was used to evaluate comorbidities. The CCI includes both the number and severity of a resident′s medical conditions. A higher score indicates a greater burden of comorbidities [27].

Evaluation of the stage of the dementia was performed according to the CDR [28]. Participants were divided into categories of having very mild/mild dementia (CDR 0.5–1), moderate dementia (CDR 2), and severe dementia (CDR 3) according to this measurement. Cognition was assessed by the MMSE [29]. The resident’s dependence in physical functioning was assessed by the CDR “personal care” question (1 = totally independent; 2 = needs prompting; 3 = requires assistance in dressing, personal hygiene, and keeping of personal belongings; 4 = requires much help with personal care, repeatedly incontinent). CDR “personal care” ≥ 2 was defined as dependence in physical functioning.

The MNA was used to evaluate nutritional status. The MNA is a validated nutrition screening and assessment tool [30]. The MNA has a maximum score of 30 points; <17 points indicates malnutrition, 17–23.5 indicates risk of malnutrition, and >23.5 indicates good nutrition status [30,31]. In addition, each resident was weighed and body mass index (BMI) was calculated as weight divided by height squared (kg/m^2^).

Energy and protein intake was determined by a one to two day food record collected by ward nurses. The food records were analyzed by using AivoDiet dietary software (version 2.2.0.0, Aivo Oy, Turku, Finland). The AivoDiet software includes the Fineli Food Composition database Release 16 (2013). The database includes recipes for typical Finnish mixed dishes that are usually served in long-term care. The instruction was to record all the foods and beverages consumed by the resident. Nurses estimated portion sizes by household measures. For prepacked products, the exact brand and product name was given. A suitable recipe was used during data entry.

Nutrition care was assessed with several questions addressed to ward nurses. The consumption of snacks was determined as follows: “Does the resident eat snacks between meals?” (yes/no). The amount of offered food eaten by the resident was evaluated with the following question: “How much does the resident on average eat from the main meal?” with the five options “eats only a little”, “eats less than half”, “eats half their meal”, “eats most of their meal”, or “eats all or nearly all of their meal” available as responses. The responses “eats only a little” and “eats less than half” were dichotomized as eats less than half and the responses “eats half their meal, “eats most of their meal”, or “eats all or nearly all of their meal” were considered as eats more than half. Use of nutritional supplements was surveyed (yes/no). The nurses were also asked whether the participant ate normal (normal or soft) food or liquid/pureed food (yes/no). The presence of symptoms such as constipation and chewing problems were inquired with yes/no questions.

The 15D instrument was used to evaluate the resident′s HRQoL. This instrument is a validated, generic measure for HRQoL [32]. The dimensions of 15D are mobility, vision, hearing, breathing, sleeping, eating, speech, excretion/elimination, usual activities, mental function, discomfort and symptoms, depression, distress, vitality, and sexual activity. The 15D can be completed during a discussion with the resident but also completed by a proxy who knows the patient well. The 15D combines the advantages of a 15-dimension profile and a single-index measure [32]. A score of 0 indicates the poorest HRQoL and 1 indicates perfect HRQoL. The 15D index is reliable, sensitive, and responsive to change [32]. If the subject was unable to respond due to poor cognition, the nurse most familiar with the resident was interviewed about the 15D.

### 2.3. Statistical Analysis

The statistical significance for the unadjusted hypothesis of linearity across categories (quartiles) of standardized energy intake and characteristics of the study participants were evaluated using the Cochran-Armitage test for trend, analysis of variance (ANOVA), and logistic (ordinal) models with an appropriate contrast. The relationship of the HRQoL (15D) at different cognition levels as the function of the standardized energy intake per day was evaluated using the analysis of variance with an appropriate contrast. A bootstrap method was used when the theoretical distribution of the test statistics was unknown or in case of violation of assumptions (e.g., non-normality). Partial correlations were calculated using 15D dimensions and standardized energy intake adjusted for age, Charlson score, and BMI. The normality of variables was evaluated using the Shapiro–Wilk W test. The Stata 15.1 (StataCorp LP; College Station, Texas, TX, USA) statistical package was used for the analysis.

## 3. Results

The study population consisted of 538 older long-term care residents of whom 78% were female. Table 1 shows the characteristics of the study population according to CDR. Of the residents, 28% had very mild or mild dementia (CDR 0.5–1), 38% had moderate dementia (CDR 2), and 34% had severe dementia (CDR 3) (Table 1). 

The residents with severe dementia were younger, more often exhibited dependence in activities of daily living (ADL) functioning, and had lower Charlson comorbidity index and lower MMSE scores than residents with very mild/mild or moderate dementia. 

The residents with severe dementia more often had a lower BMI and malnutrition than those with moderate to mild dementia. There were no significant differences in energy intake or protein intake (g/kg) between the CDR groups. Approximately 80% of the residents received snacks between meals in all CDR groups. In addition, residents with severe dementia more often received nutritional supplements and liquid/pureed food. There was no difference between groups in the proportions of those eating snacks. However, residents with severe dementia ate on average less during their meals and more often had chewing problems.

The mean (SD) 15D index was 0.65 (0.11) among residents with very mild/mild dementia, 0.60 (0.10) among residents with moderate dementia, and 0.52 (0.10) among residents with severe dementia. The difference was significant (*p* < 0.001).

**Figure 1:**
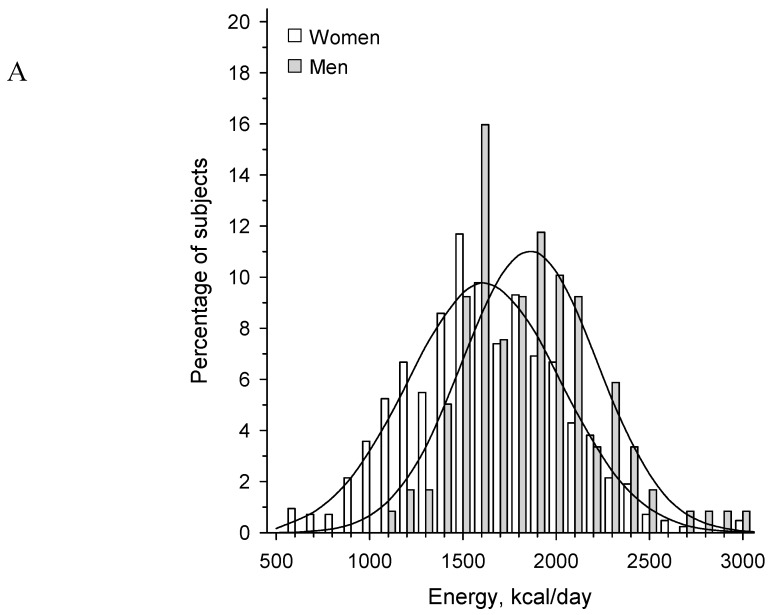

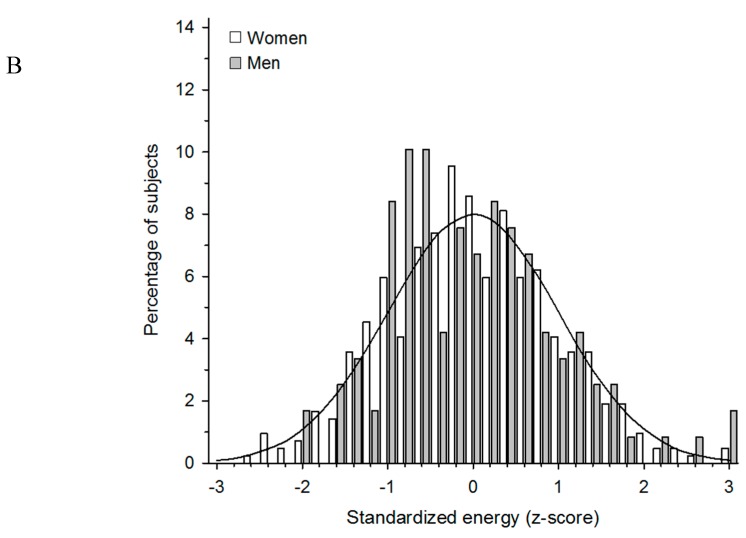
Distribution with normal curve overlay of energy intake/day in women and men (**A**) and standardized (*z*-score) energy intake/day (**B**). Energy intakes were standardized by *z*-scores to include both men and women in the same analyses.

**Figure 2:**
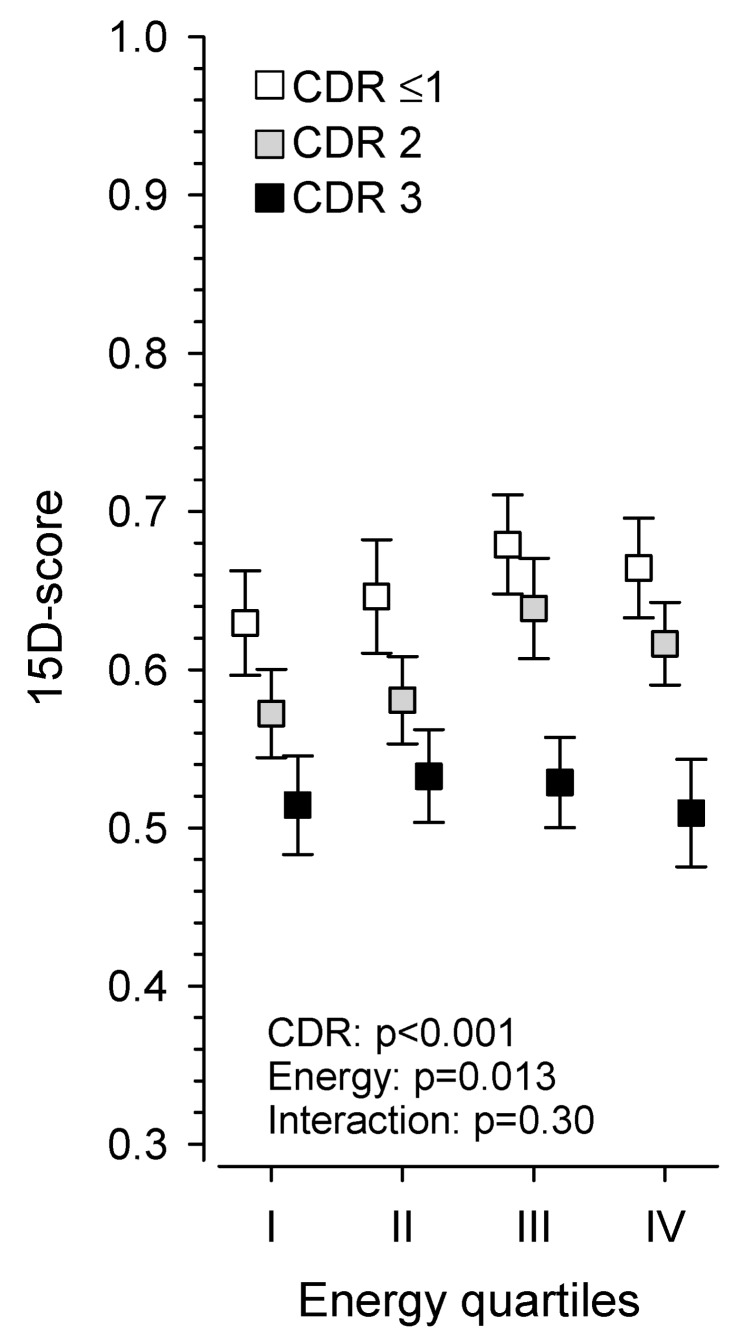
Relationship between the standardized energy intake per day at various stages of dementia according to the Clinical Dementia Rating (CDR) [28] with health-related quality of life (15D) [32].

Figure 1 panel A presents energy intake per day in males and females, and panel B shows the standardized energy intake per day (*z*-scores). Figure 2 shows the correlation between standardized energy quartiles and HRQoL in different stages of dementia. Although both stage of dementia (*p* < 0.001) and energy intake (*p* = 0.013) were significantly associated with HRQoL (Figure 2), the interaction was not significant (*p* = 0.30). 

**Figure 3:**
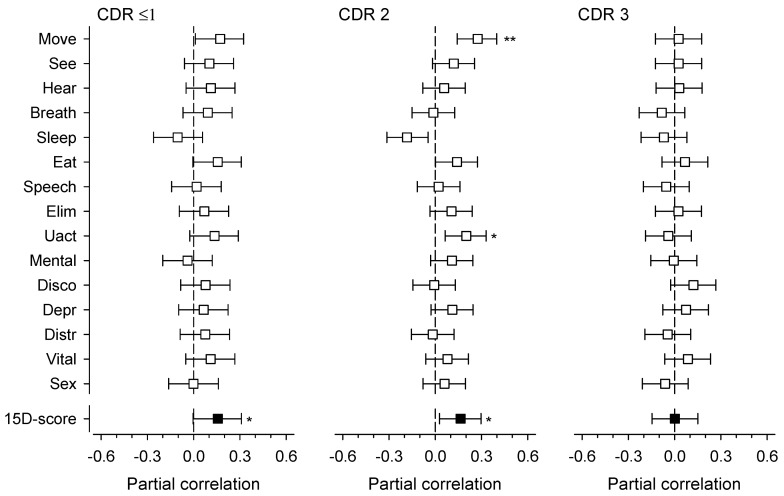
Partial correlations of standardized energy intake per day with health-related quality of life according to 15D [32] at various stages of dementia according to the Clinical Dementia Rating (CDR) [28]. The correlations were adjusted for age, Charlson Comorbidity Index [27], and body mass index. * *p* < 0.05, ** *p* < 0.01.

Figure 3 presents partial correlation analysis between standardized energy intake per day and dimensions of 15D according to CDR groups. Among residents with very mild/mild dementia, none of the dimensions of 15D were associated with standardized energy intake. In contrast, the total 15D index was significantly associated with higher standardized energy intake (Figure 3). In residents with moderate dementia, the dimensions of mobility and usual activities and the 15D index were significantly associated with standardized energy intake. Among residents with severe dementia, no significant associations emerged between the different dimensions or the 15D index and standardized energy intake.

## 4. Discussion

We observed that the stage of dementia according to CDR and standardized energy intake were both associated with HRQoL. The interaction between the stage of dementia and standardized energy intake was not significant. The partial correlation between the 15D index and standardized energy intake was significant in very mild to moderate dementia but not in severe dementia. 

The strength of this study is the large sample size concerning food records. The assessment tools are well-validated and widely used internationally. The food records were collected by trained nurses. Food records with direct observation have been considered more appropriate than food frequency questionnaires in institutional settings [33]. We included consecutive long-term care residents with mild to severe stages of dementia. We assessed the resident’s HRQoL with the 15D tool that can been completed by proxy and may thus also be used among residents with severe dementia. One limitation is that this study is cross sectional and causality cannot be determined between cognitive impairment and HRQoL or standardized energy intake and HRQoL. In addition, we did not explore the contents and servings of the foods, which may be even more important than energy and protein intake with regards to HRQoL.

In our study, residents with severe dementia had poorer HRQoL than residents with moderate or very mild/mild dementia. Our findings are consistent with some previous studies [34,35,36] but not with all [37]. In addition, few studies have included people with severe dementia. For example, Abrahamson et al. excluded nursing home residents with the most severe dementia [38]. This large American study suggested that residents with more impaired cognitive impairment reported lower QoL in several domains, including activities, individuality, privacy, and meaningful relationships. Comparisons across studies may not be possible due to the various instruments used to assess HRQoL and stage of dementia, and the differing study populations (community samples or from various long-term care settings). However, it seems that HRQoL, at least its functional dimensions, is associated with the stage of dementia.

In our study, standardized energy intake was associated with better HRQoL. This finding is consistent with previous studies among older home-care patients and non-institutionalized older people [11,24]. In addition to energy intake, malnutrition stage is associated with poor HRQoL in institutionalized and community-living older people [22,25,26,39]. These results suggest that adequate energy intake and good nutritional status may lead to better HRQoL in institutionalized older residents.

We did not observe a significant interaction between HRQoL, standardized energy intake, and dementia stage. At very mild/mild and moderate stages of dementia, a higher standardized energy intake was associated with higher HRQoL. However, at the severe stage there was no such relationship. To our knowledge, there are no previous studies that have explored how energy intake modifies the association between the stage of dementia and HRQoL. According to our findings, adequate energy intake has the potential to modify the association between HRQoL and the stage of dementia, at least in mild-moderate stages.

There was a significant partial correlation between standardized energy intake and HRQoL of residents with very mild/mild or moderate dementia, but not with residents with severe dementia. In moderate dementia, the 15D dimensions of mobility and usual activities correlated significantly with higher energy intake. Both mobility and usual activities are part of ADL functions. Thus, energy intake may modify HRQoL through ADL functions. In previous studies among long-term care residents, increased energy intake has maintained ADL functions [40,41]. ADL functions are an important part of HRQoL and may explain the association between standardized energy intake and HRQoL. ADL functions tend to decrease as dementia progresses [42]. There was no correlation between standardized energy intake and HRQoL among residents with severe dementia. Among residents with severe dementia, the ability to perform ADL functions is already greatly compromised regardless of energy intake. This may explain why higher energy intake does not modify HRQoL among these residents.

We did not observe differences between the CDR groups in energy intake, although there was a larger proportion of malnutrition among those with severe dementia than among those with mild-moderate dementia. In addition, there was no difference in eating snacks between the various stages of dementia. However, residents with severe dementia were assessed to eat on average less of their food portion than those at the very mild-moderate stages. The nurses more often administered oral nutritional supplements to those with severe dementia than to those at the very mild-moderate stages. Furthermore, residents at the severe stage of dementia more often received modified (liquid/pureed food) food to maintain their energy intake than those at the very mild-moderate stages. Thus, several means to maintain energy intake were used in these long-term care settings. 

## 5. Conclusions

This study provides evidence that both standardized energy intake and stage of dementia are significantly associated with HRQoL in institutionalized older residents. Our study emphasizes that adequate energy intake may maintain HRQoL especially among residents with mild to moderate dementia, among whom food seems to modify HRQoL through mobility and ADL functions.

## Figures and Tables

**Table 1:** Characteristics of the study population according to Clinical Dementia Rating [28].

Population Characteristics	CDR 0.5–1 *N* = 150	CDR 2 *N* = 206	CDR 3 *N* = 182	*p*-Value ^1^
***Background characteristics***				
Education (≤8 years), *n* (%)	70 (50)	87 (49)	82 (53)	0.61
Age (years), mean (SD ^2^)	85 (7)	84 (8)	83 (7)	0.002
Female (%)	116 (77)	166 (81)	137 (75)	0.60
Dependence in ADLs ^3^, *n* (%)	132 (88)	193 (94)	178 (99)	<0.001
Charlson ^4^, mean (SD)	2.3 (1.2)	2.1 (1.3)	1.9 (1.1)	<0.001
MMSE ^5^, mean (SD)	14.8 (5.5)	11.6 (5.7)	7.6 (6.2)	<0.001
***Nutrition status***				
Mean weight, kg (SD)	69 (15)	68 (15)	63 (11)	<0.001
BMI ^6^, mean (SD)	26.1 (4.8)	26.0 (5.3)	23.8 (4.2)	<0.001
MNA ^7^, *n* (%)				<0.001
Malnourished (<17 points)	12 (9)	30 (17)	46 (28)	
At risk of malnutrition (17–23.5 points)	91 (68)	124 (69)	109 (67)	
Normal nutritional status (>23.5 points)	30 (23)	26 (14)	8 (5)	
***Nutrition intake***				
Energy total kcal, mean (SD)	1684 (434)	1674 (419)	1648 (383)	0.41
Energy total kcal, mean (SD)				
Women	1611 (432)	1629 (415)	1599 (384)	0.79
Men	1936 (344)	1860 (391)	1797 (341)	0.093
Protein g/kg BW/d, mean (SD)	0.91 (0.32)	0.89 (0.30)	0.91 (0.31)	0.97
***Nutrition care***				
Eats snacks between meals, *n* (%)	116 (79)	163 (80)	142 (81)	0.70
Receives oral nutritional supplements, *n* (%)	24 (16)	45 (22)	54 (30)	0.003
Eats normal food, *n* (%)	119 (80)	125 (61)	68 (38)	<0.001
Eats less than half of the food portion, *n* (%)	35 (23)	44 (22)	26 (15)	0.046
***Symptoms related to eating and digestion***				
Chewing problems, *n* (%)	23 (16)	56 (30)	78 (45)	<0.001
Constipation, *n* (%)	44 (32)	54 (29)	45 (28)	0.45
***HRQoL***				
15D index ^8^, mean (SD)	0.65 (0.11)	0.60 (0.10)	0.52 (0.10)	<0.001

^1^*p*-value for linearity was evaluated by using the Cochran-Armitage test for trend and analysis of variance with an appropriate contrast; ^2^ SD = Standard deviation; ^3^ ADL = Activities of daily living measured by Clinical Dementia Rating (CDR) scale “personal care” score ≥ 2 [28];^4^ Charlson comorbidity index [27]; ^5^ MMSE = Mini-Mental State Examination [29]; ^6^ BMI = body mass index (kg/m^2^); ^7^ MNA = Mini Nutritional Assessment [30]; ^8^ [32]. Italic blackening in the table makes the table clearer.
